# Transcript changes in *Vibrio cholerae* in response to salt stress

**DOI:** 10.1186/s13099-014-0047-8

**Published:** 2014-12-30

**Authors:** Xiuping Fu, Weili Liang, Pengcheng Du, Meiying Yan, Biao Kan

**Affiliations:** State Key Laboratory for Infectious Disease Prevention and Control, National Institute for Communicable Disease Control and Prevention, Chinese Center for Disease Control and Prevention, 155, Changbai Road, Changping, Beijing 102206 China; Collaborative Innovation Center for Diagnosis and Treatment of Infectious Diseases, Hangzhou, 310006 China

**Keywords:** *Vibrio cholerae*, Salt stress, Transcription, PCR array

## Abstract

*Vibrio cholerae*, which is a serious human intestinal pathogen, often resides and thrives in estuaries but requires major self-regulation to overcome intestinal hyperosmotic stress or high salt stress in water and food. In the present study, we selected multiple O1 and O139 group *V. cholerae* strains that were isolated from different regions and during different years to study their salt tolerance. Based on the mechanisms that other bacteria use to respond to high salt stress, we selected salt stress-response related genes to study the mechanisms which *V. cholerae* responds to high salt stress.

*V. cholerae* strains showed salt-resistance characteristics that varied in salt concentrations from 4% to 6%. However, group O1 and group O139 showed no significant difference in the degree of salt tolerance. The primary responses of bacteria to salt stress, including Na^+^ exclusion, K^+^ uptake and glutamate biosynthesis, were observed in *V. cholerae* strains. In addition, some sigma factors were up-regulated in *V. cholerae* strains, suggesting that *V. cholerae* may recruit common sigma factors to achieve an active salt stress response. However, some changes in gene transcript levels in response to salt stress in *V. cholerae* were strain-specific. In particular, hierarchical clustering of differentially expressed genes indicated that transcript levels of these genes were correlated with the degree of salt tolerance. Therefore, elevated transcript levels of some genes, including sigma factors and genes involved in peptidoglycan biosynthesis, may be due to the salt tolerance of strains. In addition, high salt-tolerant strains may recruit common as well as additional sigma factors to activate the salt stress response.

**Electronic supplementary material:**

The online version of this article (doi:10.1186/s13099-014-0047-8) contains supplementary material, which is available to authorized users.

## Introduction

The ability to survive and thrive in severe environments is a prerequisite for pathogenic microorganisms to cause infectious diseases. Microorganisms must be able to adapt to environmental changes or stresses, such as temperature, pH and osmolarity pressure; for example, food-borne bacteria must adapt to high salt stress. The mechanisms that microorganisms use to respond to high salt stress have been investigated using model microorganisms such as *Escherichia coli*, *Bacillus subtilis* and *Shewanella* [1-5].

Generally, the mechanisms that bacteria use to respond to hypertonic stress include Na^+^ ion exclusion and K^+^ ion uptake as well as the accumulation of compatible solutes, such as glutamate. Bacteria also recruit sigma factors that recognize specific promoter regions and that guide the assembly of RNA polymerase to regulate the expression of stress-response genes [6-9]. In addition to these common mechanisms, bacteria also use several unique mechanisms to adapt to high salt stress. For example, transcript levels of chemotaxis-related genes are up-regulated in *Desulfovibrio vulgaris* Hildenborough, while those of chemotaxis-related genes are down-regulated or unaffected in *Shewanella* MR-1 [4,10]. In addition, critical enzymes involved in both aerobic and anaerobic respiration are significantly up-regulated in *Shewanella* MR-1 and *D. vulgaris* Hildenborough, while critical enzymes necessary for the tricarboxylic acid (TCA) cycle are down-regulated in *Shewanella algae* 2736 [4,10-12].

*Vibrio cholerae*, which is a serious human intestinal pathogen, often infects humans through contaminated water or food and will encounter many types of high salt stress during its life cycle, including hyperosmotic stress in the intestine and high salt stress in water and food. Therefore, *V. cholerae* requires major self-regulation to overcome these stresses.

In this study, we examined changes in the expression of genes which have been suggested to be related to salt stress response, especially those involved in iron transport, amino acid biosynthesis and peptidoglycan biosynthesis, as well as sigma factors. We selected 8 *V. cholerae* strains that were isolated from different regions and different years to study their salt tolerance and common mechanisms by which they respond to high salt stress. We analyzed transcript levels of salt stress-response related genes by using real-time PCR array.

## Materials and methods

### Strains

*V. cholerae* strains used in this study were listed in Additional file 1: Table S1. All 8 *V. cholerae* strains, including 4 serogroup O1 strains and 4 serogroup O139 strains, were toxigenic and carried *ctxAB* cholera toxin genes. These strains were isolated from different sources, such as patients or water, and from different regions in China during different years (Additional file 1: Table S1).

### Growth conditions

To identify the highest salt concentration that *V. cholerae* can tolerate, all 8 *V. cholerae* strains were cultivated overnight in LB broth (1% tryptone, 0.5% yeast extract) for use as seed cultures. Seed cultures were diluted at 1:100 and then cultivated at 37°C and 200 rpm in triplicate in LB broth containing different amounts of NaCl (0.5%, 2%, 4%, 5%, or 6%). Growth rates were measured spectrophotometrically (OD_600_) once an hour.

### Sample preparation and total RNA extraction

*V. cholerae* seed cultures were cultivated in triplicate in LB broth (1% tryptone, 0.5% yeast extract, 0.5% NaCl) for 5 h until reaching OD_600_ = 1.0. Next, 40 ml of culture solution was harvested by centrifugation (5500 rpm at 4°C for 10 min) and resuspended twice in PBS. Then, cultures were cultivated in LB broth containing 0.5% (served as control sample) and 5% NaCl.

After 1 h, total RNA was extracted using RNAiso reagent (TaKaRa), and genomic DNA was removed by incubation with DNase I (Ambion). Subsequently, the integrity of RNA was verified by analyzing intact ribosomal RNA bands using agarose gel electrophoresis, and the purity of RNA was determined using UV-spectrophotometry.

Each RNA sample (1 μg) was reverse transcribed to cDNA using SuperScript III first-strand synthesis system (Invitrogen) according to the manufacturer’s instructions. Biological triplicates cDNAs of each *V. cholerae* strain were mixed equally, and the mixture was used as a single qRT-PCR template.

### Real-time quantitative PCR (qRT-PCR)

We selected 53 salt stress-response genes encoding sigma factor, glutamate biosynthesis, peptidoglycan biosynthesis and ion-transport proteins. Specific primers for each gene were designed using primer 3 batched run by a Perl script [13] and 109–168 bp products were amplified (see Additional file 2: Table S2 for primer details).

Fluorescence PCR amplifications were performed using SYBR Green EX Taq mix (TaKaRa) in a Bio-Rad CFX96 Real-Time PCR system. The relative transcript level of each gene (cultures cultivated in LB broth containing 5% NaCl) was determined by calculating 2^-ΔΔct^ compared with the transcript level of control sample (cultures cultivated in LB broth containing 0.5% NaCl) using *gyrB* or *thyA* gene as internal control. ddH_2_O was used as negative control for qRT-PCR. These genes were then used for GO term functional analysis and KEGG pathway analysis. Each experiment was conducted in triplicates.

### The internal control gene

To identify the most stable internal control gene, 6 housekeeping genes were selected and evaluated using geNorm software. M values were calculated according to method mentioned in previous studies [14,15]. Primers for housekeeping genes amplification were designed according to previous study [16].

## Results

### Growth curves for 8 *V. cholerae* strains under different NaCl concentrations

**Figure 1:**
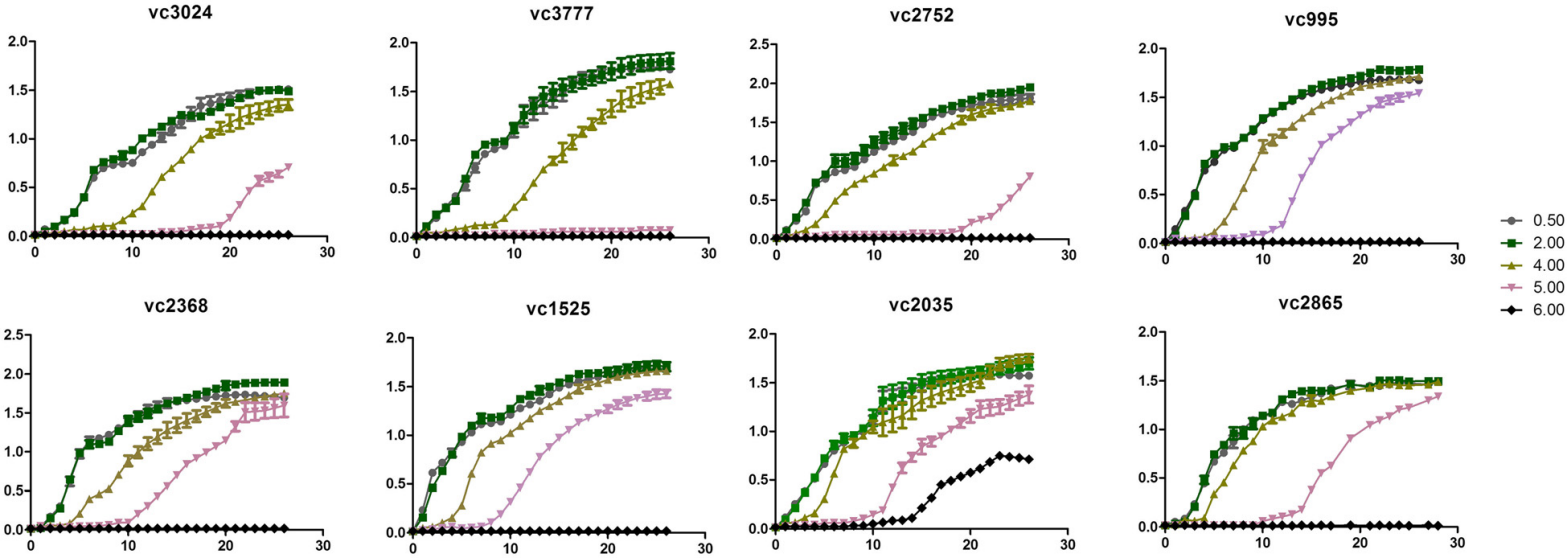
**Growth curves for the 8** ***V*** *.* ***cholerae*** **strains grown in LB broth.** VC2865 cells were cultured in LB broth containing 0.5%, 2%, 4%, 5%, or 6% NaCl.

To determine the maximum salt concentration that *V. cholerae* strains can tolerate, 8 strains were cultivated in LB broth containing different concentrations of NaCl (0.5%, 2%, 4%, 5%, 6%). Growth curves indicated that most of the strains could replicate in the presence of 0.5% to 5% NaCl. Strain VC3777 barely replicated in the presence of 5% NaCl; however, strain VC2035 replicated with a NaCl level of 6%. Relative to the other strains, strains VC 3024 and VC2752 showed a noticeable growth delay before reaching log phase in the presence of 5% NaCl. All of the 8 *V. cholerae* strains required NaCl levels between 0.5% and 2% for optimal growth. When exposed to the highest salt concentration, all 8 *V. cholerae* strains replicated slowly at first and entered stationary phase at a lower optical density (OD) than that when exposed to lower NaCl concentrations. Strain VC3777 barely replicated in 5% NaCl. Growth rates of strains VC3024 and VC2752 decreased significantly compared with those of the other strains in 5% NaCl. In addition, strain VC2035 replicated in 6% NaCl, while the other strains only replicated in the presence of 0.5% to 5% NaCl (Figure 1). According to the degree of salt tolerance, three strains (VC3024, VC3777 and VC2752) were classified as the low salt-tolerant group, and five strains (VC2368,VC995,VC1525,VC2865,VC2035) were classified as the high salt-tolerant group. O1 and O139 showed no significant difference in the degree of salt tolerance. In our study, 5% NaCl was selected to analyze salt stress responses of *V. cholera*.

### Stability evaluation of six candidate reference genes

Relative gene transcript level should be normalized to that of a stable internal control gene. Therefore, in this study, we used gene stability measure M and geNorm algorithm to identify the most stable internal control gene of 6 selected housekeeping genes. All M values of the 6 candidate genes were less than the cut-off value of 1.5 which is recommended by geNorm. The stability ranking of 6 candidate genes in 8 *V. cholerae* strains was different. According to the result of M values, *gyrB* (M < 0.35) was the most stable gene for 7 of the *V. cholerae* strains, while *thyA* (M < 0.05) was the most stable gene for strain VC1525. In addition, the mean Ct values of *gyrB* and *thyA* genes varied between 21.29 and 27.89, consistent with the recommended geNorm Ct values of 22–28. In summary, we chose *gyrB* as the internal control gene for normalization of relative transcript levels for all of the *V. cholerae* strains except for strain VC1525, for which *thyA* was used as the internal control gene (Additional file 3: Table S3).

### Transcriptional changes in *V. cholerae* in response to salt stress

We selected 53 genes, including sigma factor genes as well as genes involved in glutamate biosynthesis, iron transport and peptidoglycan biosynthesis. Total RNA was extracted using RNAiso reagent and then submitted to agarose gel electrophoresis. Clear bands of 16S rRNA and 23S rRNA were observed. The OD260/OD280 ratios of RNA samples were between 1.8 and 2.0, indicating the high integrity and purity of RNA (Additional file 4: Figure S1). Relative transcript levels were normalized to the level of *gyrB* gene or *thyA* gene.

Transcript level changes of these 53 genes in response to salt stress were strain-specific but with some common characteristics (Additional file 5: Table S4). Genes encoding the large and small chains of glutamate synthase and those encoding the transporter system *trkH* (GL0042) were up-regulated in all 8 strains. Genes encoding the proton/glutamate symporter (*gltP*, GL1168 and GLA0088) and sodium/glutamate symporter (*gltS*, GLA0041) were down-regulated in most of the strains (6/8). Transcript levels of sigma factors were not the same in 8 strains. Three sigma factors (GL0330, regulator of sigma D; GL0706, sigma-54 modulation protein; GL2302, RNA polymerase sigma-70 factor) were up-regulated in all 8 strains, and some other sigma factors, such as GL0706 (sigma-54-modulation protein), GL1522 (sigma-54-dependent response regulator), and GL1817 (sigma-54-dependent transcriptional regulator) were up-regulated in most of the strains. Some sigma factors were up-regulated in a portion of the strains but down-regulated in another portion of the strains, e.g., *rpoS* and *rpoH* (GL0150 and GL0534). In addition, changes in transcript levels of genes involved in peptidoglycan synthesis were not identical in 8 strains.

**Figure 2:**
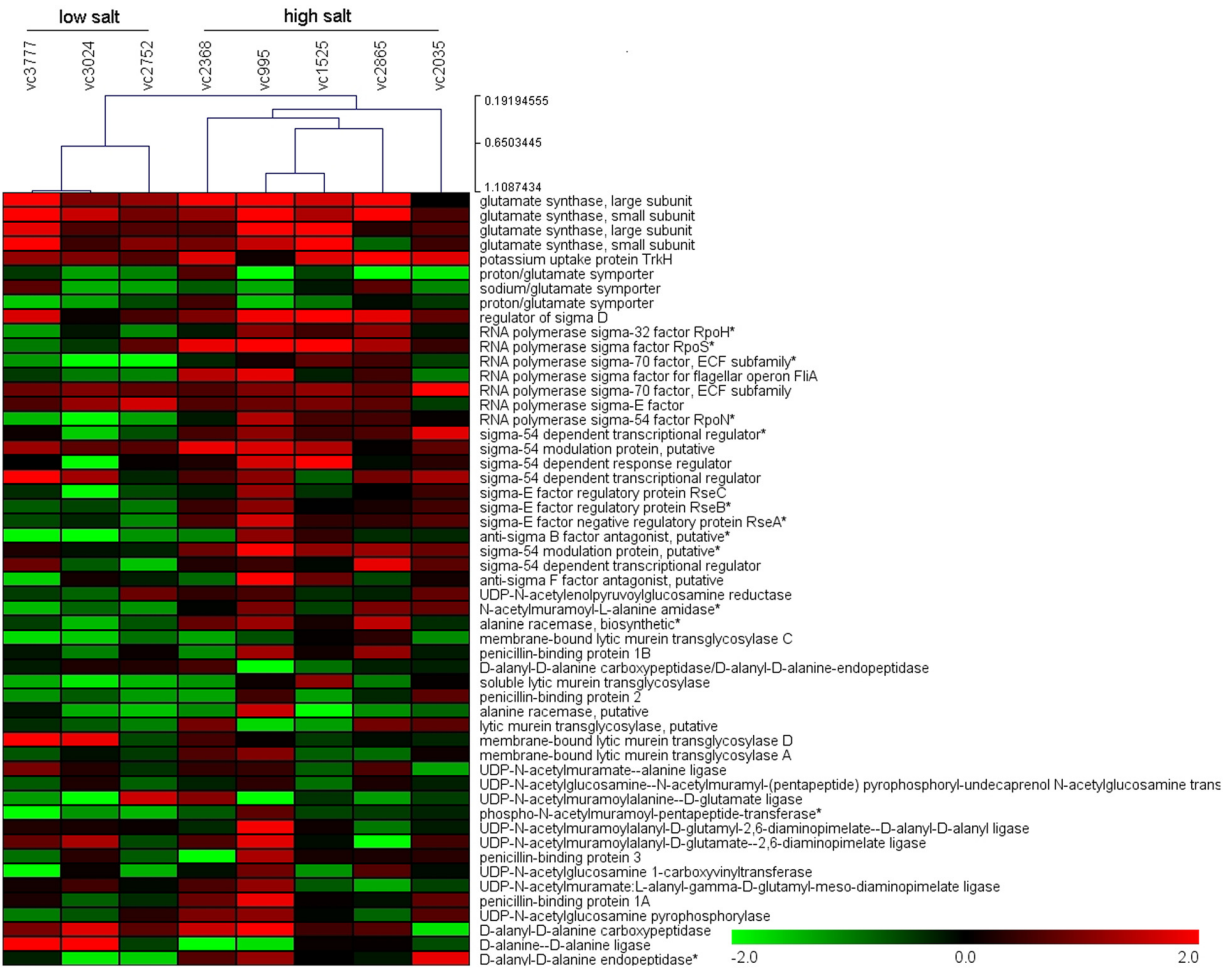
**Gene expression in response to salt stress.** Relative transcript levels of specific genes were determined by comparing the 2^-ΔΔct^ value to that in cells grown in LB containing 0.5% NaCl. The clusters were created using TMEW. Hierarchical clustering of differentially expressed genes in 8 *V. cholerae* strains.

Gene transcript profiles clustered by HCL (Hierarchical Clustering) showed that strains in the low salt-tolerant group (VC3024, VC3777 and VC2752) were clustered into one group, and strains in the high salt-tolerant group (VC2368, VC995, VC1525, VC2865, and VC2035) were clustered into another group (Figure 2). Transcript levels of these 53 genes were then analyzed using independent *t*-tests. As a result, transcript levels of 13 genes, including nine sigma factors (GL0665, GL2465, GL2466, GL2516, GL2530, GL0150, GL0534, GL1045 and GL2529) and four genes involved in peptidoglycan biosynthesis (GL0372, GL0870, GL0344 and GL2404), were significantly different between the high salt group and the low salt group. Moreover, transcript levels of these 13 genes in the high salt group were greater than those in the low salt group (Additional file 5: Table S4).

## Discussion

In this study, we explored transcript level changes of salt-tolerant genes in *V. cholerae* exposed to elevated NaCl by using real-time PCR array. Eight *V. cholerae* strains, including 4 serogroup O1 strains and 4 serogroup O139 strains, were used in the present study. These strains were isolated from different sources, such as patients, stool, and water (Additional file 1: Table S1). Our data showed no difference in the degree of salt tolerance between O1 group and O139 group.

Transcript levels were normalized to a stable internal control gene, which is necessary for qRT-PCR research. For each *V. cholerae* strain, 6 housekeeping genes were selected and evaluated using geNorm software. Housekeeping genes in 8 *V. cholerae* strains showed different expression stability. *gyrB* was the most stable gene for 7 strains, while *thyA* was the most stable gene for VC1525 strain. *GyrB* or *thyA*, with the lowest M value, was selected as the internal control gene for *V. cholerae* strains in our study. In addition, the Ct value of the internal control gene ranging from 21.29 to 27.89, was similar to those of target genes. In summary, the internal control gene selected by geNorm software was important for normalization and standardization. Thus, it is better to screen the internal control gene from 5–6 housekeeping genes for qRT-PCR array.

Eight *V. cholerae* strains in our study showed different salt-resistance characteristics, with the highest level of salt tolerance of 4% to 6% NaCl. However, no significant difference was observed in the degree of salt tolerance between group O1 and group O139. Changes in transcript levels of salt stress-response genes in *V. cholera* were strain-specific, indicating that the microbial stress response system is complicated.

Some common mechanisms were also found in the 8 strains of *V. cholerae* resisting to salt stress. The primary responses of bacteria to salt stress included Na^+^ exclusion and K^+^ uptake. Additionally, bacteria also accumulated glutamate to neutralize the increased number of cations [9]. In bacteria, three diverse K^+^ transporter systems, *Kup*, *Trk*, and *Kdp*, maintain the desired concentration of internal K^+^ [17-19]. In our study, genes encoding the *trkH* transporter system were up-regulated in each of the 8 strains. However, genes encoding the symporters *gltP* and *gltS*, responsible for simultaneously transferring one glutamate and one H^+^/Na^+^ into the cell, were down-regulated in all of the *V. cholerae* strains, which was consistent with the strategy used by other bacteria (such as *E. coli*) in response to salt stress [20]. These data indicated that Na^+^ exclusion and K^+^ uptake are the most important responses to high salt stress in *V. cholera*e. Genes encoding the large and small chains of glutamate synthase were up-regulated in all of the 8 strains, which indicated that glutamate biosynthesis is a primary mechanism used to counter salt stress in *V. cholerae*. In contrast, importing glutamate from the external environment is one of the primary mechanisms used to counter salt stress in *S. algae* [11]. In addition, three sigma factors, including the regulator of sigma D, the sigma-54 modulation protein and the RNA polymerase sigma-70 factor, were up-regulated in all of the *V. cholerae* strains. Thus, these sigma factors are important for the response of *V. cholerae* to salt stress. Furthermore, the regulator of sigma D may up-regulate the expression of sigma-38 (such as *rpoS*) and of some stress-dependent genes by sequestering sigma-70 from their promoters [21,22]. In bacteria, the RNA polymerase sigma-70 factor plays important roles during various stress responses, especially in responses to osmotic pressure [23-26].

In our study, gene transcript profiles clustered by HCL showed that strains in the low salt-tolerant group were divided into one group and strains in the high salt-tolerant group were divided into another group, which suggested a correlation between gene transcript levels and the degree of salt tolerance. *t*-test analysis showed that transcript levels of 13 genes were significantly different between the high salt group and the low salt group. Moreover, transcript levels of the 13 genes in the high salt group were greater than those in the low salt group. We considered that the elevated transcript levels of these genes might be related to the high salt tolerance of strains. Transcript levels of sigma factors *rpoS*, *rpoH* and *rpoN* were up-regulated in high salt-resistant strains. Under normal growth conditions, the *rpoS* sigma factor is largely expressed in stationary phase, while under environmental stress, such as hyperosmosis, low pH, and heat shock, the level and activity of *rpoS* increases rapidly, regulating approximately 10% of genome genes [6,27,28]. In *E. coli*, *rpoH* plays an important role during various stress responses, positively regulating more than 20 heat shock proteins when the bacterium encounters harsh environmental stress [29,30].

It has been suggested that peptidoglycan layer thickening is not a common mechanism used by *V. cholerae* in response to salt stress. However, genes encoding peptidoglycan biosynthesis were variably regulated in different strains in response to salt stress. Specifically, four genes involved in peptidoglycan biosynthesis displayed greater transcript levels in the high salt group than those in the low salt group, suggesting that the elevated expression of these genes may improve the salt tolerance of strains. In a previous study, key genes involved in peptidoglycan biosynthesis were also shown to be up-regulated in *S. algae* [18].

In conclusion, *V. cholerae* use some common mechanisms to respond to high salt stress. For example, the *trkH* gene encoding K^+^ transport protein, and genes encoding the large and small chains of glutamate synthase were up-regulated. In addition, *V. cholerae* may recruit some of sigma factors, such as the RNA polymerase sigma-70 factor, to activate the salt stress response pathway. Furthermore, strain-specific mechanisms also exist in *V. cholerae*, and transcript levels of some of these genes were correlated with the degree of salt tolerance. For example, transcript levels of the sigma factors *rpoS* and *rpoH* were elevated, indicating that the expression of these genes may improve the salt tolerance of strains. Thus, high salt-tolerant strains may recruit common sigma factors, as well as additional sigma factors , to active salt stress-response related genes.

## Additional files

Additional file 1: Table S1. Background information of *V. cholerae* strains used in this study. Additional file 2: Table S2. qRT-PCR primers. Additional file 3: Table S3. The most stable reference genes for 8 *V. cholerae* strains under high salt condition. Additional file 4: Figure S1. Analysis of RNA by agarose gel electrophoresis. Additional file 5: Table S4. Raw data and complete list of up- and down-regulated genes.
