# Supplementary material for: Transcript changes in Vibrio cholerae in response to salt stress
Source: Gut Pathog. 2014 Dec 30;6:47. doi: 10.1186/s13099-014-0047-8 (PMC4293811; doi:10.1186/s13099-014-0047-8)
Supplement: Additional file 1: Table S1. — Background information of V. cholerae strains used in this study. [file 13099_2014_47_MOESM1_ESM.docx]

**Supplementary Table S1** Background information of *V. cholerae* strains used in this study

| Sample | Serotype | Toxity | Yr of isolation | Province | Source |
| --- | --- | --- | --- | --- | --- |
| VC2865 | O1 | toxigenic | 1961 | GuangDong | Stool |
| VC3024 | O1 | toxigenic | 1973 | XinJiang | Unknown |
| VC2752 | O1 | toxigenic | 1981 | AnHui | Stool |
| VC1525 | O1 | toxigenic | 1994 | SiChuan | Patient |
| VC3777 | O139 | toxigenic | 1993 | XinJiang | Patient |
| VC2368 | O139 | toxigenic | 1999 | JiangSu | Patient |
| VC995 | O139 | toxigenic | 2001 | FuJian | Water |
| VC2035 | O139 | toxigenic | 2006 | GuangXi | Enviroment |
