# Supplementary material for: Transcript changes in Vibrio cholerae in response to salt stress
Source: Gut Pathog. 2014 Dec 30;6:47. doi: 10.1186/s13099-014-0047-8 (PMC4293811; doi:10.1186/s13099-014-0047-8)
Supplement: Additional file 3: Table S3. — The most stable reference genes for 8 V. cholerae strains under high salt condition. [file 13099_2014_47_MOESM3_ESM.docx]

**Supplemental Table S3** The most stabile reference genes of eight strains under high salt conditions

| Strain | Vc3024 | Vc3777 | Vc2752 | Vc995 | Vc2368 | Vc1525 | Vc2035 | Vc2865 |
| --- | --- | --- | --- | --- | --- | --- | --- | --- |
| Most stabile genes | recA | rpoA | recA | thyA | recA | recA | thyA | 16s |
|  | gyrB | gyrB | gyrB | gyrB | gyrB | thyA | gyrB | gyrB |
