# Supplementary figures and images for: Transcript changes in Vibrio cholerae in response to salt stress
Source: Gut Pathog. 2014 Dec 30;6:47. doi: 10.1186/s13099-014-0047-8 (PMC4293811; doi:10.1186/s13099-014-0047-8)

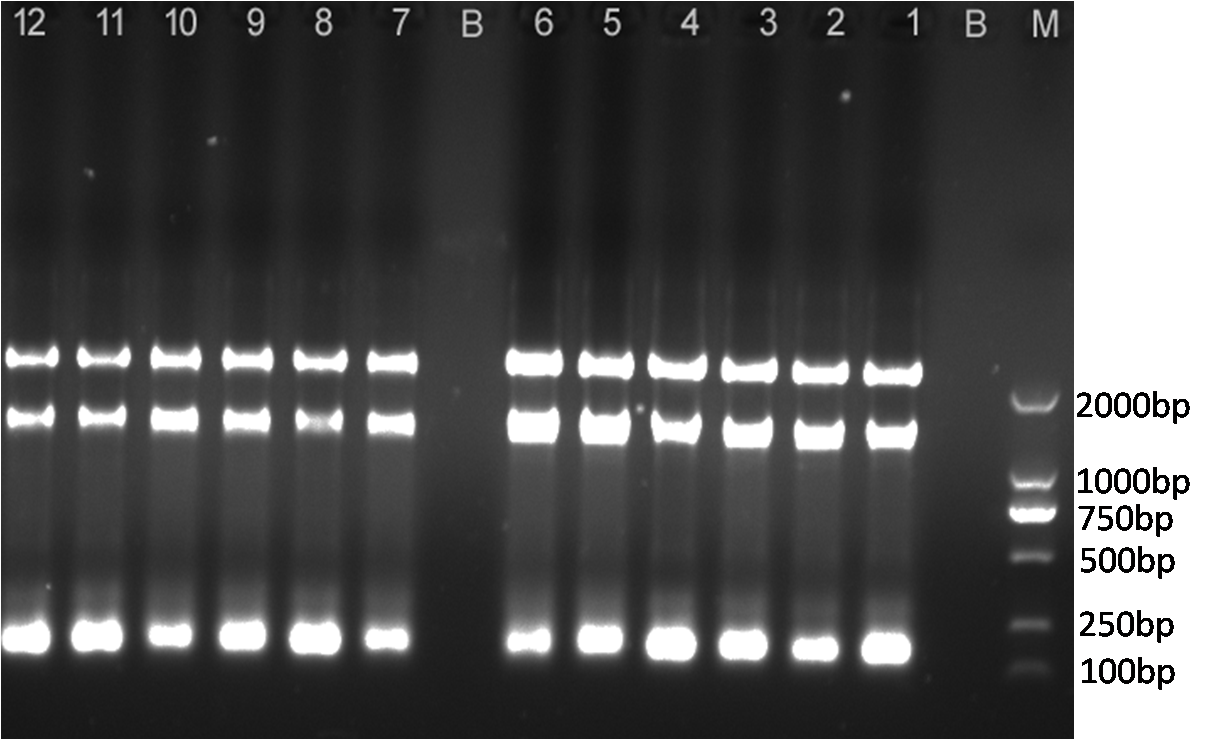

Supplement: Additional file 4: Figure S1. — Analysis of RNA by agarose gel electrophoresis. [file 13099_2014_47_MOESM4_ESM.tiff]
